# A cross-sectional study of national outpatient gastric acid suppressant prescribing in the United States between 2009 and 2015

**DOI:** 10.1371/journal.pone.0208461

**Published:** 2018-11-30

**Authors:** Hannah Bustillos, Kelsey Leer, Amanda Kitten, Kelly R. Reveles

**Affiliations:** 1 College of Pharmacy, The University of Texas at Austin, Austin, TX, United States of America; 2 Pharmacotherapy Education and Research Center, The University of Texas Health San Antonio, San Antonio, TX, United States of America; University of Mississippi Medical Center, UNITED STATES

## Abstract

**Purpose:**

Gastric acid suppressants are commonly used in the United States, and while generally well-tolerated, long-term use has been associated with infection, bone fractures, and nutrient malabsorption. The purpose of this study was to describe national trends in gastric acid suppressant use over a 7-year period.

**Methods:**

This was a cross-sectional study using data from the National Ambulatory Medical Care Survey from 2009 to 2015. Gastric acid suppressant use was defined as any outpatient visit with a documented prescription for a proton pump inhibitor or histamine-2 receptor antagonist documented during the outpatient visit. Sample data weights were used to extrapolate to national estimates. Use was calculated as the number of prescriptions per total outpatient visits per year. Appropriateness of prescribing was assessed using FDA-approved indications listed in each visit.

**Results:**

These data represent 6.8 billion patient outpatient visits between 2009 and 2015, of which nearly 600 million (8.8%) had documented gastric acid suppressant use. The median (IQR) age of gastric acid suppressant users and non-gastric acid suppressant users was 62 (50–73) and 49 (25–65), respectively. Gastric acid suppressant use decreased from 9.0% in 2009 to 7.7% in 2012, and then increased to 9.7% in 2015. Proton pump inhibitor use was slightly higher in the Midwest (8.3%). Only 15.8% of gastric acid suppressant users had a documented indication.

**Conclusions:**

Proton pump inhibitor use increased after 2012, and the majority of gastric acid suppressant users did not have a documented indication. Judicious gastric acid suppressant prescribing needs to be exercised, especially in the context of new safety data regarding long-term proton pump inhibitor use.

## Introduction

Gastric acid suppressants (GASs), including proton pump inhibitors (PPIs) and histamine-2 receptor antagonists (H2RAs), are therapeutic agents used for gastrointestinal disorders such as gastroesophageal reflux disease (GERD) and peptic ulcer disease (PUD). In the inpatient setting, these agents may also be used in critically ill patients for stress ulcer prophylaxis. H2RAs and PPIs were first introduced to the market in the 1980’s, and since then, these medications have become some of the most commonly used drug classes in the United states [1]. PPIs became especially prevalent between 1995 and 2006, with PPI treatment (43.9 prescriptions per 1000 visits) greatly outpacing the increase in GERD diagnoses (at 16.3 per 1000 visits) during that time period, likely due to their availability over-the-counter [2]. Between 2002 and 2009, the number of outpatient visits with documented PPI use more than doubled, from 30 million to 84 million; however, no documented gastrointestinal complaints or diagnoses were found in over 60% of these visits [3]. H2RA use is common as well. A 2015 study found that roughly 30 out of every 1,000 adult patients among 5 million Medicaid members had a prescription for a H2RA [4].

More recently, a significant body of evidence links long-term use of PPIs with serious adverse effects. Studies have shown that PPI use can lead to increased risk of bone fracture [5], vitamin and mineral deficiencies [6], and *Clostridium difficile* infection [7]. PPIs have also been associated with pneumonia [8], dementia [9], gastric cancer [10], and chronic kidney disease [11]. H2RAs are not as strongly linked to adverse effects like *Clostridium difficile* infection and dementia [12], but safety information on this drug class is also lacking [13].

Despite the emergence of important safety information regarding GASs, it is unknown if outpatient GAS prescribing rates have decreased in recent years. The aim of this study was to examine trends in GAS prescribing rates, as well as appropriateness of prescribing using nationally-representative outpatient data.

## Materials and methods

### Study design and data source

This was a cross-sectional study using data from the Centers for Disease Control and Prevention’s National Ambulatory Medical Care Survey (NAMCS) from 2009 to 2015.The NAMCS is an annual, national probability sampling of outpatient office visits to non-federally employed physicians engaged in direct patient care [14]. The sample includes ambulatory patients seen by a physician, physician assistant, or nurse practitioner. Patients seen by a provider in a hospital, nursing home, patient’s home, or other extended-care institution were excluded from sampling.

The NAMCS collects physician and patient demographics, along with clinical information specific to each visit. Chronic conditions are specified as such within reasons for visit and denoted within diagnosis codes. Three diagnoses, based on the International Classification of Diseases, Ninth Revision, Clinical Modification (ICD-9-CM), were recorded per visit. Up to eight medications, including prescription medications, over-the-counter medications, and immunizations that are either ordered, supplied, administered, or continued at the time of visit were also recorded. Regions of practice (Northeast, Midwest, South, and West) correspond to the U.S. Bureau of the Census geographic regions.

### Study definitions

All sampled patients from 2009 to 2015 were eligible for this study and were stratified as GAS users and non-users (S1 File). GAS use was defined as any outpatient visit with a documented order for a PPI (dexlansoprazole, esomeprazole, lansoprazole, omeprazole, pantoprazole, rabeprazole) or H2RA (cimetidine, famotidine, nizatidine ranitidine). Appropriate GAS use was defined as an ICD-9-CM code for heartburn (787.1), dyspepsia (536.8), or for a U.S. Food and Drug Administration (FDA)-approved indication for GAS use: gastrointestinal ulcer (531–534, V21.71), erosive esophagitis (530.1–2), GERD (530.81), *H*. *pylori* (0.41.86),and Zollinger-Ellison syndrome (251.5). We also completed a sensitivity analysis which included long-term non-steroidal anti-inflammatory drug (NSAID) use (V5864), esophageal stricture/stenosis (530.3), or documented NSAID or steroid use. NSAIDs included aspirin, celexoxib, diclofenac, diflunisal, etodolac, ibuprofen, indomethacin, ketoprofen, ketorolac, meloxicam, nabumetone, naproxen, oxaprozin, piroxicam, salsalate, sulindac, and tolmetin. Steroids included betamethasone, dexamethasone, hydrocortisone, methylprednisolone, prednisone, or prednisolone.

### Data and statistical analysis

Sample data weights that account for selection probability, non-response, and other factors were used to extrapolate sample counts to national estimates of ambulatory visits in the U.S. Demographics and regional prescribing were compared between GAS users and non-users using the Wilcoxon rank-sum test for continuous data and the chi-square test for nominal data. The Wilcoxon rank sum test was chosen because all continuous variables were non-normally distributed (p<0.01) using the Shapiro-Wilk test. The overall proportions of specific PPIs and H2RAs prescribed were also observed.

Prescribing rates were calculated as the number of visits with documented GAS use per total outpatient visits per year. Regional prescribing rates were calculated as the number of visits with documented GAS use in each region divided by the total number of visits in that region. The region-specific denominator was chosen because each region has a different population size, which would impact total outpatient visits in each region.

## Results

The data represent 6.8 billion patient visits to ambulatory care clinics between 2009 and 2015 (average of 965 million visits per year), of which nearly 600 million (8.8%) had documented GAS use. Table 1 provides an overview of patient characteristics. GAS users and non-users differed with respect to demographics, comorbidities, practitioner and practitioner specialty (p<0.0001 for all). GAS users were older than non-users (median age 62 vs. 49 years, respectively). Similarly, Medicare was more often the primary payment source for GAS users (43.6%) compared to non-users (25.3%). GAS users were found to have a higher number of chronic comorbidities when compared to non-users, with the greatest percentage differences in hyperlipidemia, arthritis, and diabetes. The majority of practitioners who prescribed GASs were physicians (98.3%). The primary practice specialty reported by GAS prescribers was primary care (56.2%).

**Table 1:** Baseline characteristics.

Characteristic^a^	GAS Users^b^ (n = 0.60 billion)	Non-Users (n = 6.16 billion)
Age (years), median (IQR)	62 (49–73)	49 (25–65)
Age ≥65 years, %	44.6	26.1
Female sex, %	60.3	58.0
Hispanic or Latino ethnicity, %	11.3	13.3
Race, %		
White	84.7	82.9
Black	10.8	11.1
Other	4.5	6.0
Primary payer, %		
Medicare	43.6	25.3
Medicaid	8.2	12.8
Private insurance	43.8	53.9
Other	4.4	8.0
Gastric acid suppressant indications, %		
Any indication	15.8	1.0
Ulcer	1.1	<0.1
Erosive esophagitis	0.7	<0.1
Gastroesophageal reflux disease	13.4	0.8
Heartburn	0.3	<0.1
*H*. *pylori*	0.3	<0.1
Dyspepsia	0.6	0.1
Other comorbidities, %		
Arthritis	21.8	13.1
Asthma	8.7	6.3
Cancer	8.8	5.8
Cerebrovascular disease	3.3	1.6
Congestive heart failure	3.2	1.6
Chronic obstructive pulmonary disease	6.2	3.1
Depression	13.4	9.4
Diabetes	19.9	11.8
HLD	34.2	16.3
Obesity	11.7	7.1
Osteoporosis	5.3	2.5
Chronic comorbidities, median (IQR)	2 (1–3)	1 (0–2)
Practitioner, %		
Physician	98.3	97.4
Physician assistant	7.0	5.1
Advanced nurse practitioner	2.4	2.2
Practice specialty, %		
Primary care	56.2	54.4
Medical care	30.3	25.6
Surgical care	13.5	20.0
Practitioner specialty, %		
General/family medicine	26.3	20.8
Internal medicine	24.7	13.2
Pediatrics	2.9	13.4
Obstetrics & gynecology	2.6	7.7
Other	43.5	44.9

^a^All comparisons significantly different between GAS users and non-users at a p-value <0.0001

^b^GAS use defined as any visit with a documented order for a proton pump inhibitor or histamine-2 receptor antagonist

**Fig 1:**
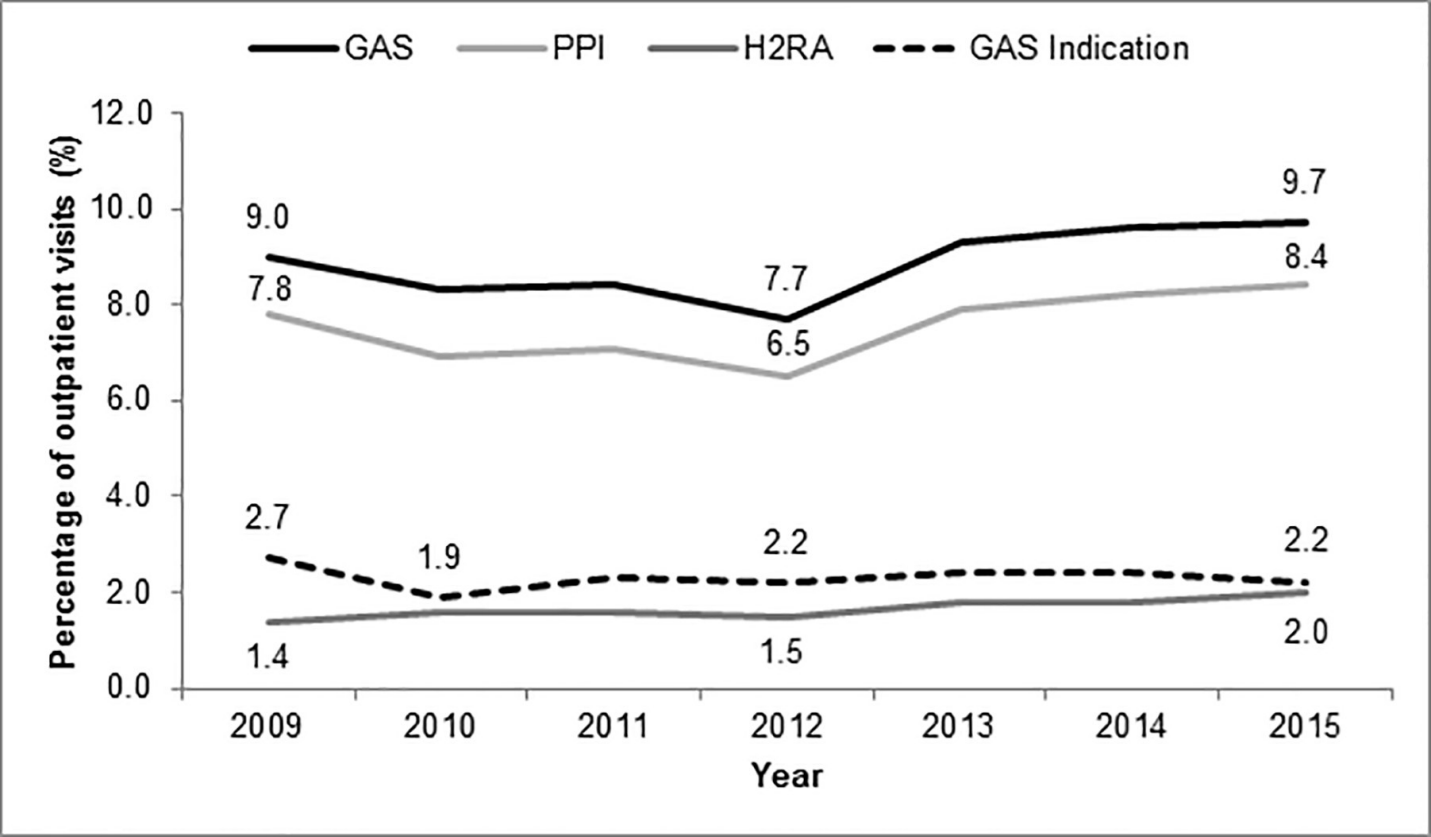
GAS prescribing and indication trends from 2009 to 2015. GAS: gastric acid suppressant; H2RA: histamine-2 receptor antagonist; PPI: proton pump inhibitor.

The most commonly prescribed PPIs were omeprazole (52.1%), esomeprazole (18.9%), and pantoprazole (14.9%); while the most commonly prescribed H2RAs were ranitidine (63.0%) and famotidine (32.7%). The percentage of PPIs prescribed per outpatient visits decreased from 7.8% to 6.5% from 2009 to 2012, but then increased to 8.4% by 2015 (Fig 1). Meanwhile, H2RA use slowly increased from 1.4% in 2009 to 2.0% in 2015. For GAS agents overall, the percentage prescribed increased from 7.7% to 9.7% of outpatient visits from 2012 to 2015. GAS prescribing was significantly higher from 2012–2015 (9.1%) compared to 2009–2011 (8.6%) (p<0.0001). These trends did not correspond to proportions of documented GAS indications, which decreased over time from 2.7% in 2009 to 2.2% in 2015.

Out of 600 million GAS users, only 15.8% were found to have a documented indication for GAS use. Meanwhile, 1% of non-GAS users had an indication. Among patients 65 years and older, only 11.4% of GAS users had an indication. The most common indication reported was GERD (84.8% of all GAS indications). A sensitivity analysis including less common indications for GAS use demonstrated that 47.2% of all GAS users and 48.3% of elderly GAS had an indication. This was primarily driven by a large proportion of GAS users who were concomitantly prescribed an NSAID (29.8%) or a steroid (5.3%). Indications for GAS use among outpatients remained relatively stable over the study period. Among GAS-users, the reason for visit was rarely for a reason likely related to GAS prescribing: heartburn (2.4%) and diseases of the esophagus (3.8%).

**Fig 2:**
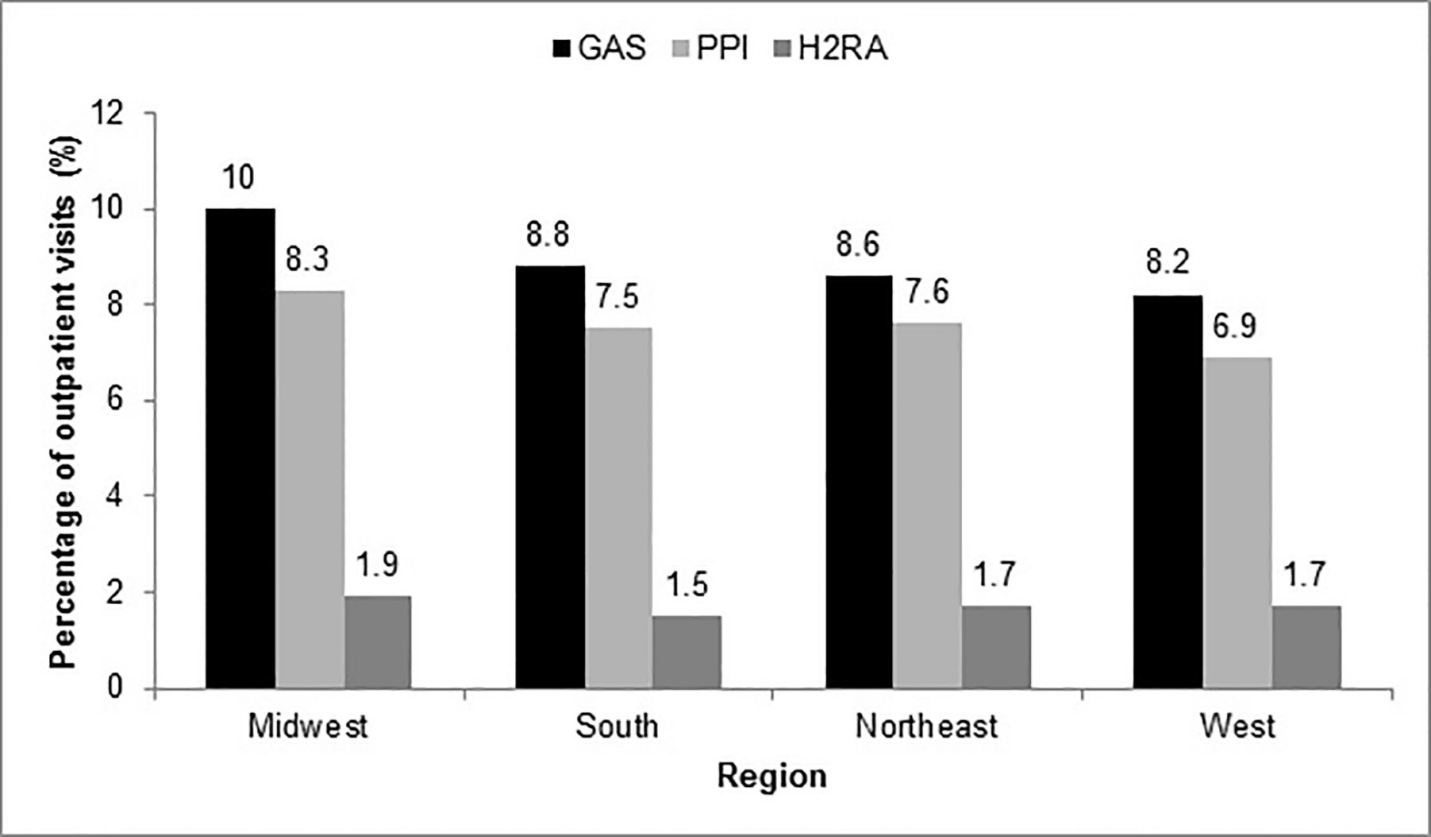
Regional GAS prescribing trends. GAS: gastric acid suppressant; H2RA: histamine-2 receptor antagonist; PPI: proton pump inhibitor.

Regional prescribing trends were also analyzed. GAS use was found to be similar across all regions, with prescribing rates slightly higher in the Midwest at 10%, compared to 8.6% in the South, 8.8% in the Northeast, and 8.2% in the West (Fig 2).

## Discussion

This study described national GAS prescribing trends and the proportion of GAS prescriptions with a documented indication for use between 2009 and 2015. To our knowledge, this is one of the first studies to evaluate how GAS prescribing trends have changed in recent years. This is especially important in light of new safety data released regarding PPIs. This study continues to show increasing GAS prescribing. It also supports the findings of previous studies that show that the majority of PPI prescriptions are written without an appropriate indication.

While our study found an increasing rate of PPI prescribing in recent years, the rate of increase seems to be slower than in prior years. A previous study found that PPI prescribing increased by 5.2% from 2002 to 2009, [3] whereas we documented only a 0.6% increase from 2009 to 2015. The slower increase in PPI prescribing observed may be due to more recent safety data regarding PPIs. In fact, in 2010 the FDA issued a warning that PPI use had a possible increased risk of bone fractures, followed by a safety announcement in 2012 regarding the association between PPIs and *Clostridium difficile* infection [15, 16]. The timing of these safety warnings could potentially explain the decrease in prescribing of PPIs seen from 2009 to 2012. However, this decreasing trend was only short-term as PPI prescribing once again increased from 2012 to 2015. Lastly, during this time period, only one new GAS was FDA-approved (dexlansoprazole in 2009); therefore, trends likely do not reflect changes in the availability of PPIs or H2RAs.

Meanwhile, H2RA prescribing remained relatively stable between 2009 and 2015, increasing from 1.4% to 2.0%. The American College of Gastroenterology guidelines for the treatment of GERD only recommend H2RAs as conditional or adjunctive therapy, whereas PPIs are listed as the therapy of choice [17]. A 2015 study found that while H2RA prescribing did not increase in adults between 2005 and 2011, prescribing rates did increase in the pediatric population [4]. H2RAs are commonly used to treat GERD in children. As previously mentioned, H2RAs are not as strongly linked to CDI [18] and other adverse effects as PPIs; however, further research is needed to investigate the long-term safety of H2RA use.

Only 15.8% of GAS users had a proper indication documented. The proportion of non-indicated GAS use was found to be even higher in the elderly population, with only 11.4% having an appropriate indication. Non-indicated PPI use was also observed in a 2013 study, where 65% of 1100 skilled nursing facility (SNF) residents who were prescribed a PPI lacked an appropriate diagnosis [19]. Notably, the median age of GAS users in our study was 62, which means a large proportion of individuals taking GASs are elderly. This is cause for concern, as GAS agents are listed within the Beers Criteria for Potentially Inappropriate Medication Use in Older Adults. The Beers List warns against the non-indicated use of PPIs in older adults (65 years and older) due to increased risk of CDAD, bone loss and fractures. It also cites delirium as a potential adverse effect from H2Ras [20]. Overall, this pattern of over- or inappropriate prescribing has not only been observed in the U.S., but also globally, with increased PPI prescribing in France [21], Iceland [22], the United Kingdom [23], and New Zealand [24].

The widespread overutilization of non-indicated GASs could lead to increased healthcare costs. A 2018 Australian study found that PPIs constituted nearly 35% of potentially inappropriate medications (PIMs) prescribed to 541 aged care facility residents, leading to the highest PIM costs [25]. A 2010 study also showed that non-indicated PPI use accrued more than $3 million in healthcare costs over a 4 year period [26]. It would therefore benefit both patients and third-party payers to decrease inappropriate GAS prescribing. In the future, effective de-prescribing practices require further exploration. A 2017 Cochrane review of six randomized controlled trials showed low quality evidence that switching from continuous to on-demand PPI therapy may increase the risk of losing symptom control [27]. Potential options include step-down therapy as opposed to abrupt discontinuation, as well as reevaluation of diagnoses in order to optimize patient therapy [28].

Our study has potential limitations, primarily related to the retrospective study design and data source. The NAMCS database only documents up to three ICD-9-CM codes per patient. It is possible that a percentage of GAS users in this study do have an appropriate, but unreported indication for GAS use. This may partially account for the low percentage of GAS users with a documented indication reported in our study. The specific reason for GAS use, nor providers’ decision to initiate or continue GAS use, cannot be determined with this study design. Similarly, a percentage of non-GAS users in our study might be taking an undocumented PPI or H2RA. Patients who took these agents over-the-counter might not be accounted for. Additionally, we could not determine whether these agents were prescribed as on demand or continuous therapy. The duration of therapy is important to consider with GASs, as these medications are often indicated for use for a limited period of time. Despite these limitations, any misclassification of prescribing or indications would likely be similar across study years and not affect national trends derived from NAMCS. Finally, the large sample size results in very high study power for statistical comparisons. Statistically significant findings should be interpreted cautiously, as small absolute differences between groups might not be clinically significant.

## Conclusions

Outpatient GAS prescribing rates increased between 2012 and 2015. Nearly 85% of all visits with documented GAS use did not report an appropriate indication. Judicious GAS prescribing needs to be exercised, especially in the context of new safety data regarding long-term PPI use. Public health initiative should aim to decrease non-indicated GAS use.

## Supporting information

S1 File Limited dataset. (ZIP)
